# Exiting the tunnel of uncertainty: crystal soak to validated hit

**DOI:** 10.1107/S2059798322009986

**Published:** 2022-10-27

**Authors:** Mathew P. Martin, Martin E. M. Noble

**Affiliations:** a Cancer Research UK Drug Discovery Unit, Newcastle University, Paul O’Gorman Building, Framlington Place, Newcastle upon Tyne NE2 4HH, United Kingdom; Netherlands Cancer Institute, The Netherlands

**Keywords:** orthogonal assays, qualitative structure–activity relationships, fragment screening, hit validation, drug discovery, biophysical techniques

## Abstract

Crystallographic fragment screens provide an efficient and effective way to identify small-molecule ligands of a crystallized protein. Due to their low molecular weight, such hits tend to have low, often unquantifiable, affinity for their target, complicating the twin challenges of validating the hits as authentic solution-phase ligands of the target and identifying the ‘best’ hit(s) for further elaboration. In this article, approaches that address these challenges are assessed. Using retrospective analysis of a recent ATAD2 hit-identification campaign, alongside other examples of successful fragment-screening campaigns, it is suggested that hit validation and prioritization are best achieved by a ‘triangulation’ approach in which the results of multiple available biochemical and biophysical techniques are correlated to develop qualitative structure–activity relationships (SARs). Such qualitative SARs may indeed be the only means by which to navigate a project through the tunnel of uncertainty that prevails before on-scale biophysical, biochemical and/or biological measurements become possible.

## Abstract 2

A brief overview of techniques used in the hit-identification/validation stage of drug discovery is given, highlighting the importance of orthogonal measurements and triangulation in early-stage crystallographic hit validation.

## 1. Introduction

**Figure 1:**
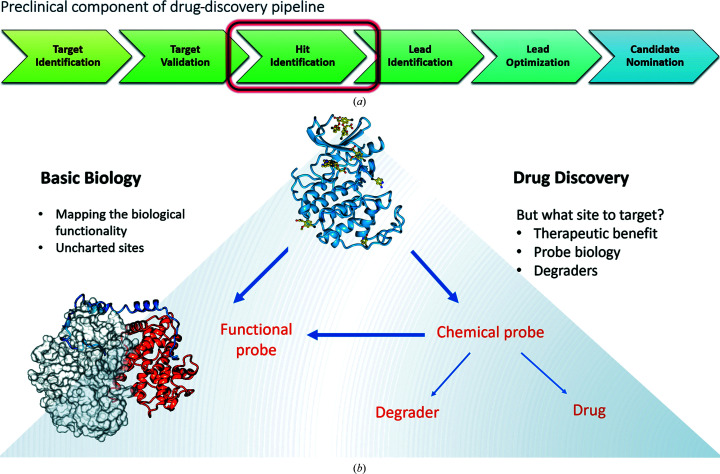
(*a*) Preclinical component of a drug-discovery pipeline. (*b*) The wealth of data that is generated from a crystallographic fragment-screening campaign can inform both basic biology and drug discovery.

Over the past 20 years, both biological and engineering advances have enabled a step change in the efficiency and effectiveness of molecularly targeted drug discovery. Each drug-discovery program arises in response to the needs of an untreatable, or poorly treated, patient population. Basic biological research identifies a molecular target or pathway that is amenable to small-molecule modulation and is expected to provide a route towards treatment of the disease with a favourable therapeutic index, *i.e.* a favourable balance between beneficial activity and deleterious side effects. The process of confirming the linkage of target modulation to the disease is referred to as target identification (TI). Once identified, a series of additional and orthogonal validation techniques are required as part of the target-validation (TV) stage before or coincident with the search for start points for drug discovery during the hit-identification (HI) stage of the drug-development pipeline (Fig. 1 ▸ *a*). Traditionally, a high-throughput screen (HTS) would be used to conduct HI. To this end, an assay suitable for high throughput would be built against the target and applied to identify a series of relatively elaborated small-molecule hits (molecular weight 350–500) with affinities typically in the micromolar range. This approach continues to be of value, although theoretical considerations suggest that it is hard to substantially cover chemical space when using libraries of molecules that have sufficient size to inhibit sufficiently potently so as to register as hits in HTS (Roughley & Hubbard, 2011 ▸; Osborne *et al.*, 2020 ▸).

More recently, HTS has been replaced or supplemented by a structural technique, such as X-ray crystallography, which provides not only the identity of the bound molecule but also a model of its bound pose (Owen *et al.*, 2016 ▸). Such is the richness of the data provided by a crystallographic screen that high-throughput crystallography can even act as part of a feedback loop to inform basic biological research during an iterative target-identification/validation process. For example, the outputs of crystallographic screens have been shown to (i) map surfaces and pockets on a target that may be relevant to biological function (Patel *et al.*, 2014 ▸), (ii) offer insight into the binding mode and interactions of known binding partners (O’Reilly *et al.*, 2019 ▸) and (iii) reveal unknown allosteric or cryptic sites which may offer novel mechanisms for target modulation (Wood *et al.*, 2019 ▸; Vajda *et al.*, 2018 ▸). Where fragments that engage with such orthosteric and allosteric sites can be brought to utility as inhibitors or degraders (Sun *et al.*, 2019 ▸), they can serve as probes of biological function (Fig. 1 ▸ *b*) and contribute further to the TI/TV process. Indeed, TI/TV can only be considered to be complete when a drug against the target has proved to be clinically beneficial, although the general expectation is that a threshold of TI/TV due diligence has been performed before HI activities are initiated.

Hits identified from a crystallographic fragment screen yield a different challenge to that presented by outputs from HTS. Fragment-based crystallographic screens present drug-discovery teams with a fragment hit (molecular weight of ∼150) of known binding mode but with (often unmeasurably) low binding affinity for the target, typically in the millimolar range (Schiebel *et al.*, 2016 ▸). Fragment binding may also be an artefact of the crystalline state of the target, for example where each molecule of the ligand forms interactions with more than one protein in the crystalline lattice. Only when the challenge of quantifying, or minimally ranking, fragment hits is overcome can quantitative structure–activity relationship (SAR) investigations be used to develop initially weak binding fragments into high-affinity modulators, an outcome which is progressed during the later stages of lead identification and lead optimization through to candidate nomination (Fig. 1 ▸ *a*). This article will provide an overview of some techniques and tools that can be used to inform this early hit-expansion phase.

## 2. Association of binding affinities with crystallographic screen hits

**Figure 2:**
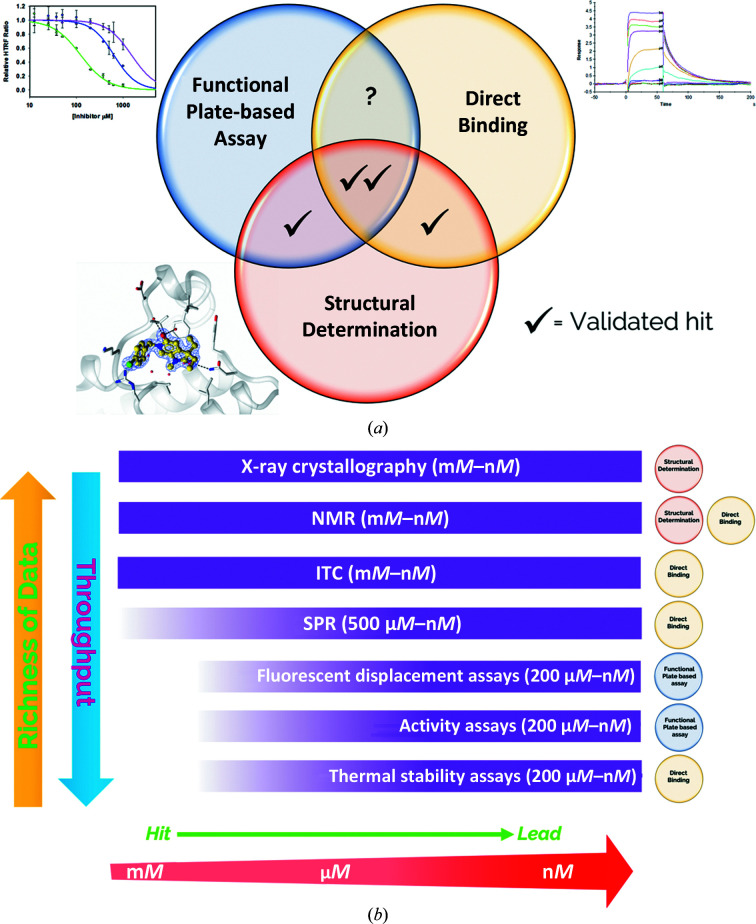
(*a*) What constitutes a validated hit: orthogonal measurements and triangulation in early-stage crystallographic hit validation. (*b*) Techniques available in the hit-validation process.

There are a wide range of techniques that may be used to assess the binding affinity of a fragment against a target (Fig. 2 ▸), with the majority of techniques falling into the categories of either functional plate-based assays or techniques to directly measure ligand binding. Each of these techniques has technical considerations that limit its ability to confidently score the binding of ‘weak’ ligands, so that a process of triangulation is indicated where the measurement of activity by multiple techniques is used to build confidence in the authenticity of each fragment hit and to assign them a relative priority for advancement into lead identification (Hughes *et al.*, 2011 ▸). Arriving at a fragment hit from a crystallographic screen implies that the major challenge is generating protein in a suitable quantity and with suitable purity for the reagent-intense techniques that may be used for orthogonal assessment (Fig. 2 ▸ *b*).

### 2.1. Nuclear magnetic resonance (NMR)

NMR can be used to monitor ligand binding by observing the environment-dependent magnetic properties of certain atomic nuclei in either the protein target or in the ligand under study. Provided that the target and ligand can be brought to sufficient concentration, NMR is capable of detecting and quantifying high-micromolar to low-millimolar affinities of fragment-binding events.

The least experimentally demanding approach is to look for a signal from the nuclei of bound ligands to which magnetization is transferred either from nuclei within the protein (in saturation transfer difference spectroscopy; STD; Mayer & Meyer, 1999 ▸) or via water molecules tightly bound to the protein (WaterLOGSY; Dalvit *et al.*, 2000 ▸). The underlying physics of magnetization transfer means that such ‘ligand-observed’ techniques are more sensitive when the target–ligand complex tumbles relatively slowly, so that binding to smaller proteins may be harder to detect.

For relatively small proteins, it is possible to observe the consequences of ligand binding in the 1D proton NMR spectrum of a protein sample at a high-micromolar or millimolar concentration. If the molecular weight of the target protein size lies towards or beyond a limit of ∼100 kDa, the spectral consequences of ligand binding can become hard to distinguish using 1D NMR approaches and it becomes necessary to use heteronuclear (2D/3D) NMR approaches to deconvolute the dense 1D spectrum. Except under optimal conditions of field strength, detector sensitivity and sample concentration, 2D and 3D protein-observed NMR techniques require protein expression to be revisited using approaches that permit the uniform or specific incorporation of isotopically enriched ^13^C or ^15^N.

Among the more widely used 2D NMR approaches used in hit validation/quantification is ^1^H–^15^N HSQC (Shuker *et al.*, 1996 ▸). By monitoring the chemical shift perturbations induced by fragment binding in an HSQC experiment, it is possible to quantitatively determine both the binding affinity and (if the HSQC spectral features have been assigned to their corresponding amino acids) the location of binding-site residues. There are other quantitative NMR methods that are used in fragment binding and these have been well studied and reviewed: Harner *et al.* (2013 ▸) give an excellent overview of NMR in fragment-based drug discovery, while a more recent review by Carneiro *et al.* (2017 ▸) focuses on NMR in structure-based drug discovery. From a practical perspective, Gossert and Jahnke have produced an excellent practical guide to identifying and validating interactions (Gossert & Jahnke, 2016 ▸).

### 2.2. Isothermal titration calorimetry (ITC)

Much like NMR, ITC has been widely used in drug discovery to characterize ligand binding (Ward & Holdgate, 2001 ▸). Being a label-free, homogenous (*i.e.* involving a single state of matter) technique, an ITC experiment has few confounding experimental variables to account for. For this reason, it is widely considered to be the gold standard for observing a ligand–protein interaction (Freyer & Lewis, 2008 ▸; Johnson, 2021 ▸). A microcalorimeter has two cells and a syringe; one of the cells is a reference cell, while the other contains the protein target sample. The calorimeter maintains the temperature of the two cells during injection of the small-molecule fragment from a sample syringe, with the heat produced during the binding event being proportional to the energy used to keep the temperature between the cells the same (Ladbury & Chowdhry, 1996 ▸). This label-free technique can be used to obtain the change of enthalpy (Δ*H*), the binding constant (*K*_d_) and the stoichiometry (*n*) of the target–small molecule fragment binding event, from which the corresponding changes in Gibbs free energy and entropy can be calculated.

One caveat of ITC is the reduced ability to accurately measure very high (picomolar) or very low (millimolar) binding affinities due to challenges in accurately measuring heat exchanges in these regimes. However, experimental design through ligand competition (Krainer & Keller, 2015 ▸) and under ‘low-*c*’ conditions (Tellinghuisen, 2008 ▸) allow ITC to be used to follow extremely tight and extremely weak binding events, respectively. Additionally, binding events which involve relatively small changes in enthalpy (*i.e.* those that are driven by favourable changes in entropy) can be hard to detect. Typically, small-molecule fragments yield a *K*_d_ in the high-micromolar to low-millimolar range towards a target. Calculating the Wiseman *c* parameter for such an interaction (*c* = *n*[fragment]/*K*_d_) would yield a *c* value of <1 for achievable concentrations of the fragment, so that the experiment is said to be run under ‘low-*c*’ conditions (Turnbull & Daranas, 2003 ▸). One of the key assumptions in designing an experiment under low-*c* conditions is the number (*n*) of binding sites. Where the ligand has initially been observed in a crystallographic screen, the number of binding sites can be readily identified. Even so, achieving a sufficient concentration of fragment to enable a quantative readout from the ITC experiment requires a solubility in the millimolar range. In general, fragment libraries are designed to enable this, with the constituent compounds having a relatively favourable solubility compared with the more elaborated compounds generally seen in HTS libraries.

### 2.3. Surface plasmon resonance

Surface plasmon resonance (SPR) is a powerful technique that can yield information about the affinity and kinetics of ligand binding, while being relatively low in protein requirement and capable of relatively high throughput. For this reason, it is widely used for hit identification and to support subsequent iterative medicinal chemistry (Myszka & Rich, 2000 ▸; Shepherd *et al.*, 2014 ▸; Navratilova & Hopkins, 2010 ▸). Although SPR may be carried out ‘label-free’ (*i.e.* using unmodified protein), the requirement to immobilize the target on the experimental dextran-coated gold SPR surface is often achieved by expressing the protein with an affinity tag. Optimization and validation of immobilization conditions for the target are the principal challenges in establishing a robust SPR assay, with immobilization typically being achieved through exploiting an affinity tag or by direct immobilization though ‘amine coupling’ of the lysine residues on the surface of the target (Wong *et al.*, 2009 ▸; Fischer *et al.*, 2011 ▸). SPR is an optical technique that detects the change in the refractive index of the immobilized target upon the SPR surface. This surface is presented on a glass support in such a way that incident polarized light generates surface plasmons, which are detected via a consequent change in refractive index, in a manner that is proportional to the mass of the sample immobilized close to the gold surface (Patching, 2014 ▸). Therefore, any change in the number of molecules bound to the surface causes a readily detected signal that can be measured in real time. This real-time monitoring allows kinetic measurement of the rate of association (*k*_a_) and the rate of dissociation (*k*_d_) of molecules from the solution phase. The *k*_d_ (‘off-rate’) can be determined from the shape of the response curve when the analyte (in this case the fragment hit) is withdrawn from the continuously flowing mobile phase. *K*_d_ is obtained from the ratio *k*_d_:*k*_a_. While the equilibrum binding constant *K*_d_ is the most important output from an SPR experiment, values of the off-rate have been used to triage libraries in early hit identification and also in lead development. Authentic binding events of very low affinity tend to be characterized by relatively fast off-rates, while for compounds characterized later in the drug-discovery process it may be those with slow off-rates that are of the greatest interest for further development (Murray *et al.*, 2014 ▸; Danielson, 2009 ▸).

Implementing a direct binding technique such as NMR, ITC or SPR to validate crystallographic data can be an extremely powerful approach in the early stages of drug discovery. There are many excellent examples in the literature where the combination of an orthogonal biophysical technique with crystallographic data has underpinned the successful further development of fragment hits (Kobayashi *et al.*, 2010 ▸; Hennig *et al.*, 2012 ▸; Navratilova & Hopkins, 2011 ▸). However, each of these techniques can generate artefacts or difficult-to-interpret results when pushed to analyse low-affinity interactions, so we have adopted an approach of ‘triangulating’ structural and biophysical data with a functional plate-based assay to deliver more robustly interpretable SARs and thereby to improve confidence in the hit-identification/validation phase.

### 2.4. Functional plate-based assay

A functional plate-based assay is typically performed in 96-, 384- or 1536-well format to provide decreased cost, lower reagent use and higher throughput. The end-point readout of the plate-based assay is most commonly light-based (for example luminescence or fluorescence), although there are excellent examples where radiometric or mass spectrometry-based end points have been used (Gale & Yan, 2015 ▸; Kategaya *et al.*, 2017 ▸). Suitable assays are designed to generate these end points in a manner that depends upon the enzymatic reaction or macromolecular interaction that the drug-discovery process is targeting. For example, to quantify the activity of a target that has kinase activity, the turnover of ATP to ADP is often measured using a luminescent or fluorescent ADP-coupled reaction (Zegzouti *et al.*, 2009 ▸) or the mass-spectroscopic detection of a phosphorylated substrate (Müller *et al.*, 2016 ▸). Where the activity that is being targeted is a protein–protein interaction there is no enzymatic turnover of product, so that the assay has to detect the proper formation of the protein–protein complex. In such cases, the interaction can be monitored through labelling the target and partner protein with an appropriately matched pair of fluorophores or with a lumigenic enzyme and a matched fluorophore, respectively. Where matched fluorophores are used, proximity can be monitored using Förster resonance energy transfer (FRET; Song *et al.*, 2011 ▸) or homogeneous time-resolved fluorescence (HTRF; Degorce *et al.*, 2009 ▸). Where a lumigen and a fluorophore are used, the proximity is measured by bioluminescent resonance energy transfer (BRET; Harikumar *et al.*, 2017 ▸). The use and the development of functional plate-based assays has been well reviewed in a number of excellent articles (Busby *et al.*, 2020 ▸; Iversen *et al.*, 2004 ▸; Roy, 2018 ▸).

### 2.5. Other techniques to consider

In addition to this arsenal of methods, there are several other excellent techniques that are worth investigating, including differential scanning fluorimetry (DSF; also known as thermal shift/thermal melt), microscale thermophoresis (MST) and bilayer interferometry (BLI). These biophysical techniques have been well reviewed elsewhere (Canales, 2017 ▸; Genick & Wright, 2017 ▸).

## 3. Consideration of the setup of the technique

The tools and techniques developed to study the affinity and inhibitory (or activatory) potency of a ligand during hit validation form the backbone of a make–test cascade in subsequent iterative SAR studies. Given the longterm advantages that derive from establishing robust assay systems at this point, the design of such assays merits careful consideration. Of prime importance is ensuring that the assay measures a quantity that can be used as a predictive surrogate for modulation of the biological system being targeted. Confirming that the assay responds appropriately when challenged with a suitable positive control, such as a ligand/inhibitor with known activity against the target, is one way to address this. However, such tool compounds are not always available and there are a number of ways to validate assay systems in the absence of a control compound.

One approach to confirming that an assay is reading out a relevant binding event is to confirm that the behaviour of the assay changes appropriately if the target is substituted by a mutated version in which the targeted site of interest is changed. Alternatively, comparing the readout of the assay when using a related and an unrelated target protein can be informative: if the same readout of hit activity is achieved irrespective of the identity of the target, there is clearly cause for concern.

During the build process it is important to ensure the robustness of any assay by monitoring a ‘measure of quality’, the nature of which is highly dependent on the technique being employed (Murray & Wigglesworth, 2017 ▸). Regardless of the technique, however, there is considerable advantage in carrying out multiple technical and biological replicates of any measurement to increase the value of the data generated. For a plate-based assay, comparison of the internal consistency of technical replicates of positive and negative control wells in any one run of an assay allows the calculation of *Z*′ (Zhang *et al.*, 1999 ▸), a statistic that takes a value in the range 0–1. An assay with a *Z*′ of greater than 0.5 is considered to be useful. Beyond the internal consistency of any one run of an assay, however, reproducibility of the technique across an extended period and using multiple batches of regent is essential to ensure the comparability of data: a drug-discovery programme may run for several years, with multiple experimenters, potentially in different laboratories, so that ensuring the reproducibility and robustness of data is paramount.

Although hit identification and validation is of relatively low cost in the grand scheme of drug discovery, there is still a cost associated with all of the techniques that must be considered during design. Instruments and their respective running costs are expensive, as are reagents. However, reducing the reagent cost by buying in bulk, aliquoting and downsizing assay formats to 384-well or 1536-well plates can help to reduce the cost considerably. Although this article does not attempt to cover small-molecule fragment chemistry and synthetic development (Murray & Rees, 2016 ▸), we must consider their effects on the assay technique. Typically, compounds are stored and/or prepared for assay by dissolution in organic solvents such as dimethyl sulfoxide. Accordingly, each assay must be validated for the concentrations of co-solvents that will be used, with close attention being paid to any interference caused by these or other buffer additives (Hughes *et al.*, 2011 ▸).

In addition to scientific determinants that underpin the choice and implementation of assays to support hit identification/validation, pragmatic considerations are also likely to apply. For example, the choice of assay may depend on the availability of technology and/or expertise locally. It may also be necessary to use a qualitative assessment of binding to prioritize chemistry effort, inferred from the performance of a compound across multiple assay formats, where more concrete quantitative data is not available. This situation arises, for example, where only incomplete binding curves can be obtained given the solubility of a hit relative to its affinity or to the sensitivity of the assay. Especially under these circumstances, communication about the strengths and weaknesses of the data that are generated is essential within a project team that will typically comprise expertise in structural biology, protein science and medicinal chemistry. For this purpose, it is also advisable to implement effective approaches to capturing and communicating results across the team.

## 4. Hit identification and validation in the ATAD2 fragment campaign

**Figure 3:**
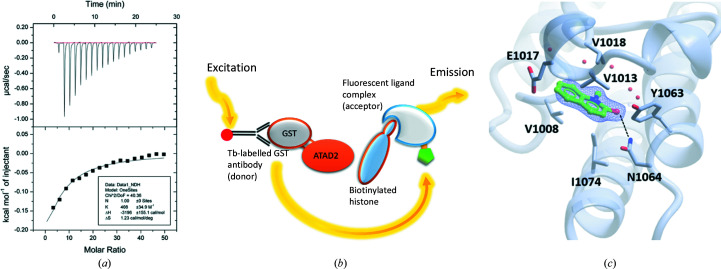
Hit identification and validation in the ATAD2 fragment campaign. (*a*) The low-*c* ITC thermogram and (*b*) the HTRF experimental assay design used in early-stage fragment hit validation. (*c*) The crystal structure of the elaborated fragment based on the initial validation results (PDB entry 7px5).

ATPase family AAA domain-containing protein 2 (ATAD2) is an epigenetic reader protein that binds to the lysine-acetylated tails of histones via a bromodomain. Researchers in the CRUK Newcastle Drug Discovery Unit undertook a probe-discovery campaign targeting the bromodomain of ATAD2, starting from four fragment series that were initially identified through a focused crystallographic screen. Given that the bromodomain of ATAD2 is expressed in high-milligram yields from a pGEX bacterial expression system, a number of techniques (Fig. 2 ▸ *a*) were readily available. The binding modes of the four fragment scaffolds, pyridinone, isoxazole, acetamide and triazole, mimicked the histone acetyllysine, each forming a single hydrogen bond to the conserved residue Asn1064. All had molecular weights under 150, and were supplied as 200 m*M* DMSO stock solutions. In an attempt to rank the series, we first applied a 384-well DSF assay using ATAD2 (5 µ*M*) and fragment concentrations of 2 m*M* in a buffer containing 5× SYPRO Orange and 1% DMSO. The melt curve for ATAD2 alone under these conditions indicated a relatively low *T*_m_ of 33.7°C, and the subsequent Δ*T*_m_ values were less than 0.5°C for each of the fragment series. At the time of the study there was no available control compound to assess the significance of these values, and therefore an additional direct binding measurement technique was investigated. Consequently, ITC was attempted under low-*c* conditions, given that the *n* value could be sourced from the crystallographic data, which indicated one binding event per compound. Fresh powder was obtained for each of the fragment series, with experiments conducted by titrating 40 m*M* fragment into 150 µ*M* ATAD2 in the cell at 25°C in matched buffer (50 m*M* HEPES pH 7.4, 200 m*M* NaCl, 1 m*M* DTT, 2% DMSO). From this set of low-*c* ITC experiments, only the fragment from the pyridinone series provided sufficient heat exchange to suggest a *K*_d_ of 2.4 m*M* (Fig. 3 ▸ *a*).

In addition to the direct binding techniques, a functional plate-based assay in the form of a 384-well low-volume HTRF assay was set up. Here, the HTRF sandwich was formed using uncleaved GST-ATAD2 (5 n*M*) and biotinylated histone H4 (500 n*M*) incubated with the excitation donor Tb-anti-GST antibody (5 n*M*) and the acceptor streptavidin-XLL65 (62.5 n*M*) in a buffer consisting of 50 m*M* Tris pH 7.5, 100 m*M* NaCl, 1 m*M* DTT, 100 µg ml^−1^ BSA, 2% DMSO (Fig. 3 ▸ *b*). To interrogate fragment binding, we performed a ten-point titration series from 4 m*M* down to 25 µ*M*, with a positive control of DMSO alone and a negative control using a point mutant of ATAD2 at the conserved asparagine (N1064A). To account for compound interference the titration series was measured in the absence of protein. The resultant assay typically yielded an excellent *Z*′ score of 0.9, which gave greater confidence in extrapolating data beyond the highest titration concentration of the fragment to guide SAR. All four fragment series yielded IC_50_ values of greater than the highest concentration measured; however, extrapolation to higher concentration was used to guide early chemistry decision points. Initial structurally guided analogues within the pyridinone fragment series were commercially purchased. One of these catalogue compounds was 1-methyl-2-quinolone, the crystal structure with which (PDB entry 7px5; Supplementary Table S1) revealed that the bicyclic ring system maintained engagement with the conserved Asn1064 and formed a hydrophobic sandwich with valines 1008, 1013 and 1018, which resulted in an on-scale IC_50_ of 2.6 m*M*, as part of an initial fragment elaboration (Fig. 3 ▸ *c*). Further validation of this compound as a crystallographic screening hit is provided by the parallel study of Chaikuad *et al.* (2014 ▸). Although those authors did not further characterize the binding of 1-methyl-2-quinolone, they did use NMR chemical shift perturbation studies to cross-correlate the binding of certain pyridmidine-containing crystallographic hits. Their use of NMR mirrors the practice in our laboratory of prioritizing hit validation by applying orthogonal binding and/or biochemical activity assays.

## 5. Discussion

**Figure 4:**
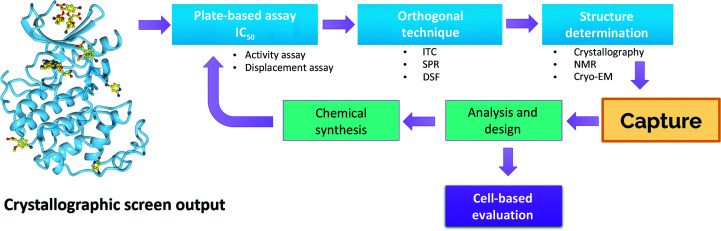
Typical workflow of a make–test cascade during hit validation and development.

Previous successful fragment-development campaigns that have resulted in preclinical compounds can provide insight into the design of an effective early-stage make–test cascade (Fig. 4 ▸). An excellent resource with which to survey such cases in fragment-based drug discovery (FBDD) is Dan Erlanson and Teddy Zartler’s Practical Fragments Blog (https://practicalfragments.blogspot.com/). One of the programmes highlighted on this site is work by Heightman and coworkers on the development of ERK1/2 inhibitors (Heightman *et al.*, 2018 ▸). Here, they combined a crystallographic screen with DSF for hit validation and subsequently drove SAR studies using a time-resolved fluorescence (TRF) activity assay. Another example presented on the Practical Fragments Blog is further work by Tamanini and coworkers targeting apoptosis protein 1 (cIAP1) and X-linked inhibitor of apoptosis protein (XIAP; Tamanini *et al.*, 2017 ▸). In this study, Tamanini and coworkers followed up crystallographic hit identification with a fluorescence polarization binding assay to support chemistry. Work by Heidenreich and coworkers targeting the YEATS domain of eleven-nineteen-leukaemia protein (ENL; Heidenreich *et al.*, 2018 ▸) used a combination of techniques to advance the crystallographic fragment hit. Initial chemical matter was sourced commercially and ITC was used extensively to support development. In another examplar, research from the Wistar Institute targeted the Epstein–Barr viral protein EBNA1, supporting medicinal chemistry around the initial fragment hit with a DNA-binding assay, SPR, ITC and 2D (HSQC) NMR (Messick *et al.*, 2019 ▸).

Research performed at AstraZeneca highlights the fragment-based discovery of an allosteric MEK1 binder (Di Fruscia *et al.*, 2021 ▸) resulting from a multi-step 1D NMR screen, with chemistry being driven through SPR. A team led by Stephen Fesik used NMR-based screening of a large fragment library to identify several chemically distinct hits that bound to the WIN site within WDR5, a chromatin-regulatory scaffold protein (Wang *et al.*, 2018 ▸). The team engaged in hit validation used crystallography alongside fluorescence polarization anisotropy and TR-FRET competition assays to drive SAR studies. It must be mentioned that fragment hits have been developed for bioactive compounds in the absence of either structural data or a reconstituted cell-free assay, and work by researchers at Merck with GPR7 showcases this nicely, wherein a cell-based assay was used to inform SAR studies (Moningka *et al.*, 2020 ▸). In summary, fragment-based drug discovery is a powerful tool and will continue to be used by the drug-discovery community in developing small molecules in response to the challenge of addressing an unmet or poorly treated patient population.

## 6. Future directions

While structurally enabled FBDD campaigns in recent years have mostly used crystallography and NMR for screening and subsequent steps, the advent of ‘the resolution revolution’ (Kühlbrandt, 2014 ▸) in cryo-electron microscopy (cryo-EM) is already having an impact in this space. Recent work by Saur *et al.* (2020 ▸) on model protein systems exemplifies how cryo-EM can be used to reveal the details of molecular interactions between fragments and protein targets. It is expected that the utility of this technique in FBDD will continue to expand as the technology advances further, addressing the current limitations of resolution, low-molecular-weight limit and long data-collection times (Van Drie & Tong, 2020 ▸).

Another technique that is becoming available to support fragment-based drug discovery is cellular thermal shift assay (CETSA), an assay that can assess the engagement of a target by a fragment in a cellular setting (Martinez *et al.*, 2018 ▸). This approach complements work from the Cravatt laboratory, in which fragment screening is conducted within human cells (Parker *et al.*, 2017 ▸; Asiaban *et al.*, 2020 ▸).

Finally, it is worth noting that both experimental design and subsequent iterative medicinal chemistry are likely to benefit from developments in artificial intelligence (AI) and machine learning (ML). The outstanding performance of AI/ML-based approaches [for example *AlphaFold*2 (Jumper *et al.*, 2021 ▸) and *RoseTTAFold* (https://robetta.bakerlab.org/)] in the recent CASP14 study (Kryshtafovych *et al.*, 2021 ▸) suggests that structures can now be predicted from primary sequences with significant confidence, streamlining the process of construct design to support implementation of all of the experimental approaches discussed above. It is likely that similar algorithms will enhance the computational design of inhibitors, steered by experimental results, to give rise to a further increase in the efficiency and effectiveness of FBDD.

## Supplementary Material

Supplementary Table S1. DOI: 10.1107/S2059798322009986/qt5007sup1.pdf
