# Supplementary material for: Exiting the tunnel of uncertainty: crystal soak to validated hit
Source: Acta Crystallogr D Struct Biol. 2022 Oct 27;78(Pt 11):1294–302. doi: 10.1107/S2059798322009986 (PMC9629488; doi:10.1107/S2059798322009986)
Supplement: Supplementary file 1 [file d-78-01294-sup1.pdf]

STRUCTURAL BIOLOGY

Volume 78 (2022)

Supporting information for article:

Exiting the tunnel of uncertainty: crystal soak to validated hit

Mathew P. Martin and Martin E. M. Noble

Table S1 Data collection, processing, structure solution and refinement of ATAD2 in complex with 1-Methyl-2-quinolone (pdb 7PX5, DOI: 10.2210/pdb7px5/pdb)

Values for the outer shell are given in parentheses.

Diffraction source	Rotating Anode
Wavelength (Å)	1.54
Temperature (K)	100
Detector	MAR scanner 300 mm plate
Space group	P 65 2 2
a, *b*, *c* (Å)	79.5, 79.5, 138.2
α, β, γ (°)	90,90,120
Resolution range (Å)	38.29-2.18 (2.25-2.18)
Rmerge	0.06 (0.39)
Total No. of reflections	32620
No. of unique reflections	13347
Completeness (%)	95.2 (83.1)
Redundancy	2.4 (2.0)
CC(1/2)	0.997 (0.724)
⟨ *I*/σ(*I*)⟩	8.4 (1.6)
No. reflections all/free	12670/632
R-factor/R-free	0.206/0.246
Cruickshank DPI	0.1815
Protein B-factor (No. atoms)	31.6 (1069)
Ligand B-factor (No. atoms)	45.1 (29)
Water B-factor (No. atoms)	36.8 (96)
R.m.s. deviations	
Bonds (Å)	0.0107
Angles (°)	1.81
